# Bismuth oxyhalides: synthesis, structure and photoelectrochemical activity†
†Electronic supplementary information (ESI) available. See DOI: 10.1039/c6sc00389c


**DOI:** 10.1039/c6sc00389c

**Published:** 2016-03-09

**Authors:** Davinder S. Bhachu, Savio J. A. Moniz, Sanjayan Sathasivam, David O. Scanlon, Aron Walsh, Salem M. Bawaked, Mohamed Mokhtar, Abdullah Y. Obaid, Ivan P. Parkin, Junwang Tang, Claire J. Carmalt

**Affiliations:** a Materials Chemistry Centre , Department of Chemistry , University College London , 20 Gordon Street , London WC1H 0AJ , UK . Email: c.j.carmalt@ucl.ac.uk; b Department of Chemical Engineering , University College London , Torrington Place , London WC1E 7JE , UK; c Bio Nano Consulting Ltd , The Gridiron Building , One Pancras Square , London N1C 4AG , UK; d University College London , Kathleen Lonsdale Materials Chemistry , Department of Chemistry , 20 Gordon Street , London WC1H 0AJ , UK; e Diamond Light Source Ltd. , Diamond House, Harwell Science and Innovation Campus, Didcot , Oxfordshire OX11 0DE , UK; f Centre for Sustainable Chemical Technologies , Department of Chemistry , University of Bath , Bath , BA2 7AY , UK; g Global E3 Institute , Department of Materials Science and Engineering , Yonsei University , Seoul 120-749 , Korea; h Chemistry Department , Faculty of Science , King Abdulaziz University , Saudi Arabia; i Surface Chemistry and Catalytic Studies Group , King Abdulaziz University , Saudi Arabia

## Abstract

We report the synthesis and photoelectrochemical assessment of phase pure tetragonal matlockite structured BiOX (where X = Cl, Br, I) films. The materials were deposited using aerosol-assisted chemical vapour deposition. The measured optical bandgaps of the oxyhalides, supported by density functional theory calculations, showed a red shift with the increasing size of halide following the binding energy of the anion p-orbitals that form the valence band. Stability and photoelectrochemical studies carried out without a sacrificial electron donor showed the n-type BiOBr film to have the highest photocurrent reported for BiOBr in the literature to date (0.3 mA cm^–2^ at 1.23 V *vs.* RHE), indicating it is an excellent candidate for solar fuel production with a very low onset potential of 0.2 V *vs.* RHE. The high performance was attributed to the preferred growth of the film in the [011] direction, as shown by X-ray diffraction, leading to internal electric fields that minimize charge carrier recombination.

## Abstract 2

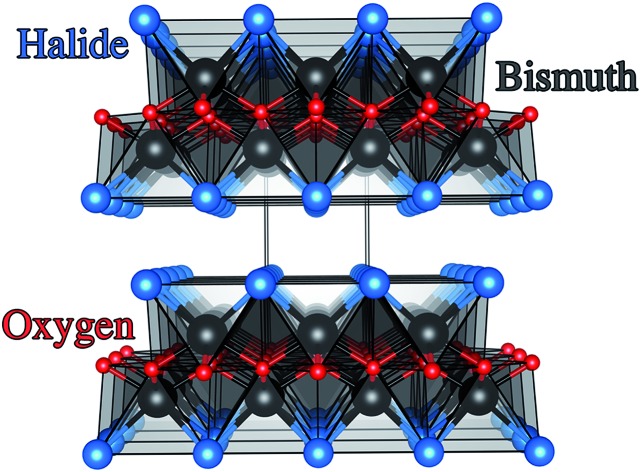
We report the synthesis and photoelectrochemical assessment of phase pure tetragonal matlockite structured BiOX (where X = Cl, Br, I) films.

## Introduction

Photoelectrochemical (PEC) water splitting was known as early as 1969 from work carried out by Fujishima and Honda on n-type rutile TiO_2_ single crystals.^1^,^2^ This finding laid the foundations for the development of materials that harvest solar energy for photocatalytic purification and solar energy conversion. The PEC process proceeds when a semiconducting material absorbs light that is equal or above the energy of its optical bandgap. This then results in electrons or holes that are transported to surface sites where redox reactions can occur. Additionally, the carriers must have sufficient chemical potential to drive water oxidation and reduction, as determined by the absolute band energies.^3^–^5^ TiO_2_ is the most studied material for this application as it not only meets the aforementioned criteria but is also highly photoactive.^6^–^8^ However, the optical bandgap of TiO_2_ at around 3.0 eV lies in the ultraviolet (UV) portion of the solar spectrum, which severely limits the maximum power conversion.^9^–^11^ As a result, PEC research of late has been heavily driven into finding visible light active materials suitable for photoelectrodes.^12^–^14^

**Fig. 1:**
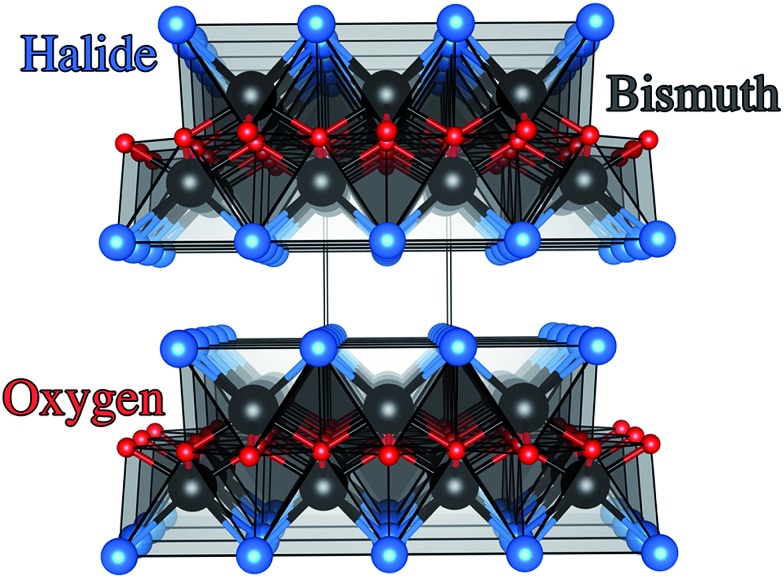
Crystal structure of the BiOX systems (space group *P*4/*nmm*, *D*_4h_ symmetry) with stoichiometric X–Bi–O–Bi–X bi-layers stacked along the *c* axis. Bismuth, oxygen and halide ions are denoted by black, red and blue spheres, respectively.

Traditional methods to visible light active materials have involved modifying wide gap semiconductors such as TiO_2_ and ZnO through doping, alloying, junction formation or sensitization in order to drive the bandgap into the visible region with varying degrees of success.^11^,^12^,^14^–^19^ The second approach has been to use well-known solar absorber materials, such as Si, GaP and GaAs that have much lower optical band gaps than TiO_2_.^13^ For example III–V semiconductor materials such as GaInP_2_ and n–p junctions of GaAs coupled to a Pt counter electrode have resulted in light conversion efficiencies of 12.4%.^13^ These materials however are quite unstable in aqueous conditions. Recent work by the Lewis group showed that unstable photoanodes such as Si, GaP and GaAs could be markedly improved in terms of stability and hole conduction by protecting the photoelectrodes with TiO_2_ layers grown by atomic layer deposition (ALD) and adding a surface electrocatalyst.^13^ More recently, solution processed tandem solar cells of CH_3_NH_3_PbI_3_ coupled to a NiFe layered double hydroxide catalyst demonstrated a solar-to-hydrogen efficiency of ∼12.3%.^20^ Material instability under working conditions however was again a significant issue. The third strategy involves developing new functional systems. These are materials that have not been conventionally used as photoelectrodes and range from simple oxides, sulphides,^21^ complex oxides and more recently metal-free materials such as graphitic carbon-nitride.^22^–^25^ Bismuth oxyhalides, BiOX (X = Cl, Br, I) are a class of V–VI–VII ternary semiconductor materials.^26^–^28^ These materials all crystallise in a tetragonal matlockite structure,^29^,^30^ which is a layered structure made up of [X–Bi–O–Bi–X] slabs stacked together by van der Waals interactions, as shown in Fig. 1. In this [X–Bi–O–Bi–X] motif, each bismuth centre is surrounded by four oxygen atoms and four halogen atoms, giving rise to an asymmetric decahedral symmetry. The combination of strong intralayer covalent bonding coupled with interlayer van der Waals attractions makes these materials interesting for anisotropic structural, electrical, optical and mechanical properties.^28^ Traditionally, bismuth oxyhalides have been investigated as catalysts, ferroelectric materials, storage materials, and pigments.^31^ More recently however, these materials have been tested for a wide range of applications from photocatalytic waste water and indoor-gas purification, water splitting, organic synthesis, and selective oxidation of alcohols.^31^–^34^ These materials have shown some promise in the photocatalytic degradation of organic dyes, but very little has been reported on their PEC properties.^27^,^31^,^35^–^37^ Their potential arises from the open crystalline structure of the material coupled with an indirect band gap that reduces the probability for electron–hole recombination.^38^ In addition, the crystal structure also results in internal electric fields perpendicular to the [X–Bi–O–Bi–X] slabs.^39^ This can potentially facilitate efficient charge separation along the [001] direction, thus further suppressing recombination.^38^

In the present study, a series of BiOX (X = Cl, Br, I) films were synthesised, for the first time, using aerosol assisted chemical vapour deposition (AACVD). The films were characterised structurally using a combination of standard laboratory techniques and compared to density functional theory (DFT) calculations, which provides insights into the electronic structure of these materials. More importantly, and to gain a complete understanding of the photophysical properties of the as-deposited BiOX films, their photoelectrochemical response was analysed under 100 mW cm^–2^ illumination in 0.5 M Na_2_SO_4_ electrolyte. All films showed photoactivity, with the BiOBr sample showing the highest photocurrent ever reported for that system without the use of a sacrificial electron donor. The BiOBr film even displayed photocurrents three times higher than that recorded for a BiOBr-reduced graphene composite electrode that was tested under similar conditions by Li *et al.*^40^ Furthermore, the BiOBr film also showed the best photostability, with only limited photocorrosion after one hour of testing, compared to the BiOCl and BiOI deposited by AACVD.

## Experimental

### Synthetic procedure

**Fig. 2:**
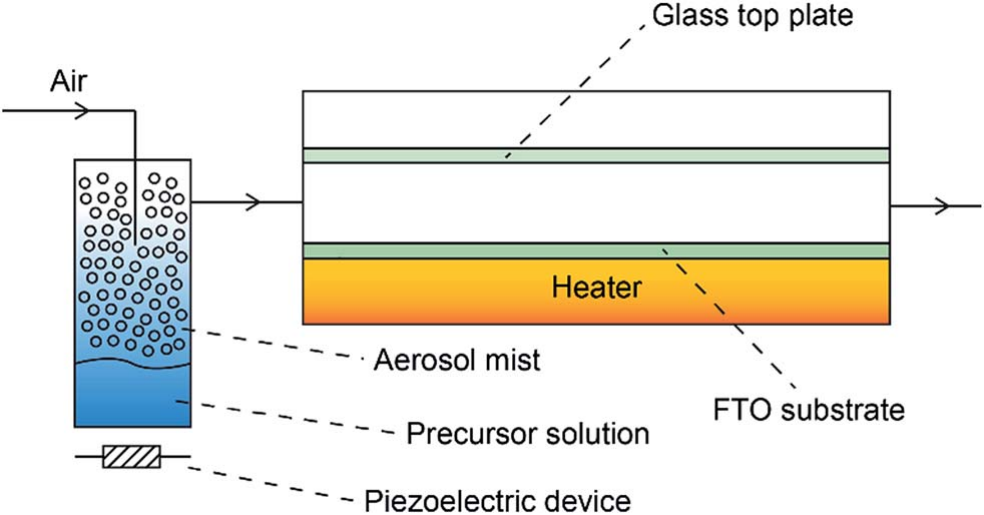
Schematic of the AACVD used for the deposition of BiOX films on FTO substrates. The aerosol mist of the precursor solution that was generated by a piezoelectric device was carried over the heated FTO substrate in the CVD reaction chamber using air.

Depositions were carried out under air. Precursors were placed in a glass bubbler and an aerosol mist was created using a piezoelectric device (Fig. 2). All chemicals were procured from Aldrich and were used as received.

BiX_3_ (X = Cl, Br, I) (1 mmol) was dissolved in *N*,*N*-dimethylformamide (25 ml). The resulting solution was stirred for 30 minutes and then atomised. The precursor flow was kept at 1 l min^–1^. The glass substrate was FTO glass, NSG TEC™ 15, 15 cm × 4 cm × 0.3 cm. A top plate was suspended 0.5 cm above the glass substrate to ensure laminar flow. The substrate was maintained at a temperature of 300 °C for a deposition time of 60 minutes. After the deposition the bubblers were closed and the substrates were cooled under a flow of air. The glass substrate was allowed to cool with the graphite block to less than 100 °C before it was removed. Coated substrates were handled and stored in air. The coated glass substrate was cut into *ca.* 1 cm × 1 cm squares for subsequent analysis.

### Film characterisation

Powder X-ray diffraction (PXRD) patterns were measured in a modified Bruker-Axs D8 diffractometer with parallel beam optics and a PSD LynxEye silicon strip detector. This instrument uses an unmonochromated Cu Kα source operated at 40 kV with a 30 mA emission current. The incident beam angle was set at 1° and the angular range of the patterns collected was 8° < 2*θ* < 66° with a step size of 0.05° counted at 0.5 s per step. Scanning electron microscopy (SEM) was performed to determine surface morphology and film thickness using a JEOL JSM-6301F Field Emission SEM at an accelerating voltage of 5 keV. Optical spectra were obtained using a Perkin Elmer Fourier transform Lambda 950 spectrometer over a wavelength range of 300 nm to 1100 nm (4.1 eV to 1.1 eV). This range embraces the ultraviolet (UV), visible and near infrared (NIR) regions. The spectra were referenced against an air background. X-ray photoelectron spectroscopy (XPS) was performed in a Thermo Scientific K-alpha photoelectron spectrometer using monochromatic Al-Kα radiation. Survey scans were collected in the range 0–1100 eV (binding energy) at a pass energy of 160 eV. Higher resolution scans were recorded for the main core lines at a pass energy of 20 eV. Valence band spectra were also recorded. Peak positions were calibrated to carbon and plotted using CasaXPS software.

### Photoelectrochemical testing

The PEC measurements were conducted in a three-electrode cell equipped with a quartz window and potentiostat (Ivium technology). The as-prepared films were used as the working electrodes. Pt mesh and Ag/AgCl (3 M KCl) were used as counter electrodes and reference electrodes, respectively. The scan speed was 20 mV s^–1^ between –0.5 and 1.5 V (*vs.* Ag/AgCl). The electrolyte was 0.5 M Na_2_SO_4_ (pH 6.5) aqueous solution, degassed with argon for 30 min. A 150 W xenon lamp (Newport, USA) equipped with an AM 1.5 G filter was used to irradiate the electrodes from the front side and was calibrated to 1 sun illumination (100 mW cm^–2^) using a photodiode. Mott–Schottky (impedance) plots were obtained at a frequency of 1 kHz in the dark with an AC amplitude of 5 mV. The flat band potential (*V*_fb_) was determined by eqn (1):1
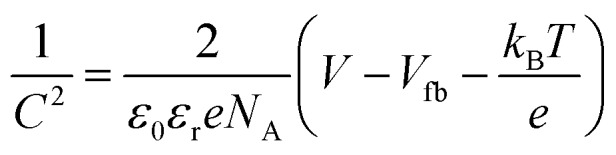
here *N*_A_ is the carrier density, *ε*_0_ is the permittivity in a vacuum, *ε*_r_ is the relative permittivity, *V* is the applied potential, *T* is the absolute temperature, *e* corresponds to the electronic charge, and *k*_B_ is the Boltzmann constant. Hence a plot of 1/*C*^2^*vs.* potential (*V*) will yield a line, which when extrapolated to the *x*-axis, will correspond to the flat-band potential of the semiconductor.^1^ Potentials were referenced to the reversible hydrogen electrode (RHE) using the Nernst equation (eqn (2)):

2 *E*_RHE_ = *E*0Ag/AgCl + *E*_Ag/AgCl_ + 0.059pH, *E*0Ag/AgCl = 0.1976 at 25 °C

### Computational methods

All electronic structure calculations were performed using the Vienna *Ab initio* Simulation Package (VASP),^41^–^44^ a periodic plane wave DFT code which includes interactions between the core and valence elections using the Project Augmented Wave (PAW) method.^45^ Both the plane wave basis set and *k*-point sampling were checked for convergence, with a cutoff of 520 eV and *k*-point grid of *Γ*-centred 6 × 6 × 2, and for the unit cell of BiOX were found to be sufficient. Geometry optimization was performed using the Heyd–Scuseria–Ernzerhof (HSE06) hybrid DFT functional^46^ with the inclusion of Grimme's D3 correction,^47^ which deals with the inability of DFT to describe weak dispersion interactions. Relativistic spin orbit effects (SOC) were included for the band structure calculations, as materials containing heavy elements such as Bi and I are known to display large relativistic renormalization.^48^–^50^

## Results and discussion

BiOX (where X = Cl, Br, I) films were deposited on FTO substrates from the AACVD reaction of BiX_3_ and *N*,*N*-dimethylformamide at 300 °C. The films were uniform, showed excellent coverage across the FTO substrate and were well adhered, passing the Scotch™ tape test.

### XRD, SEM, optical and XPS characterization

**Fig. 3:**
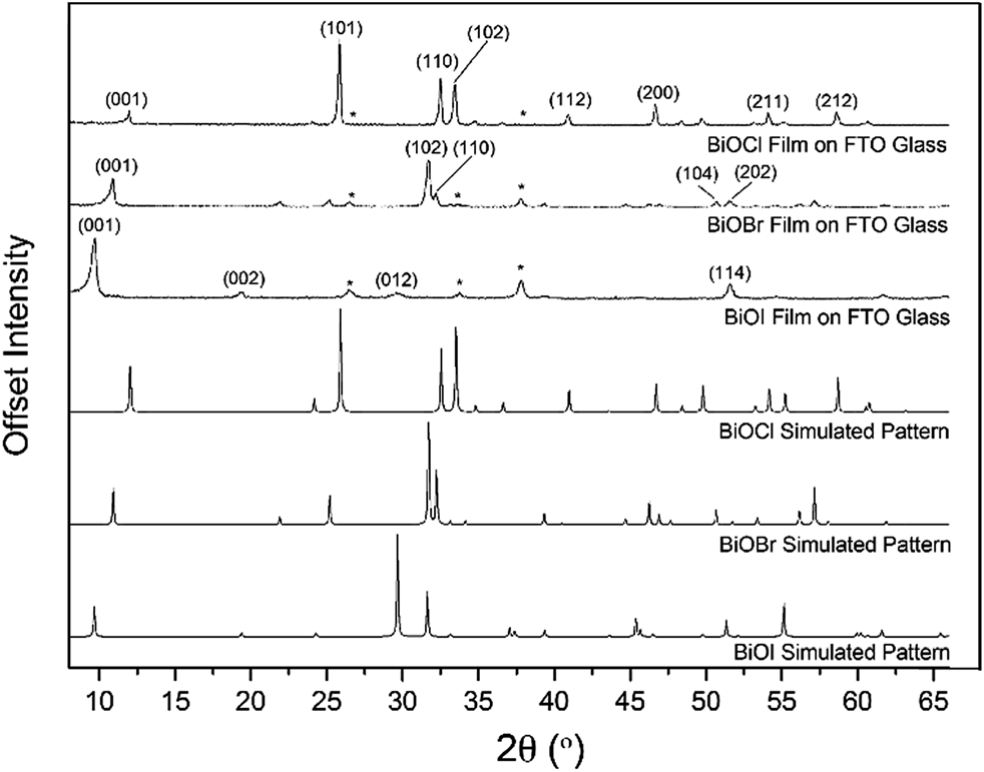
PXRD of BiOX (X = Cl, Br, I) films deposited on FTO glass from the AACVD reaction of BiX_3_/DMF in air at 300 °C along with simulated BiOX patterns. The asterisked peaks correspond to positions of reflections from the F : SnO (FTO) substrate.

X-ray diffraction patterns of the as-deposited films formed by AACVD (Fig. 3) confirmed the presence of BiOX (X = Cl, Br, I). All the films were phase pure and crystallised in the expected tetragonal matlockite structure. The BiOI films displayed pronounced (011) texture. This is in contrast to previous reports on spray deposited BiOI thin films displaying preferred (102) followed by (110) orientation.^51^ The BiOBr films exhibited (001) and (101) texture, whereas the BiOCl films showed mainly (101) and (110) texture. It has been noted before in the literature that this texture/faceted nature of the crystals may be beneficial in establishing internal electric fields aiding electron–hole charge separation in these materials.^38^

**Fig. 4:**
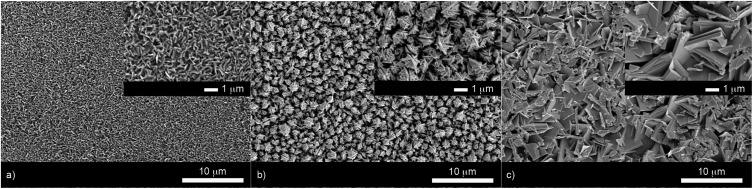
SEM images of (a) BiOI, (b) BiOBr and (c) BiOCl thin films deposited on FTO glass from the AACVD reaction of BiX_3_/DMF in air at 300 °C. Insets show a higher magnification image of the same area.

**Fig. 5:**
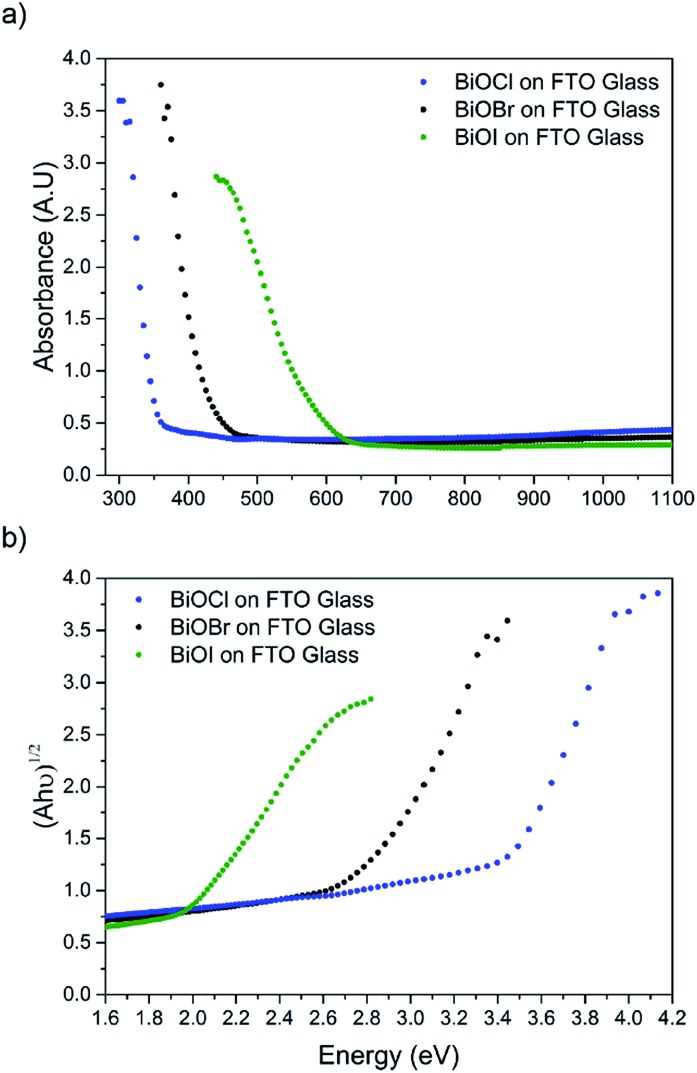
(a) UV/vis spectra of BiOX (X = Cl, Br, I) films deposited on FTO glass and (b) corresponding Tauc plots.

The film microstructure was probed using SEM as shown in Fig. 4. BiOI and BiOCl films consist of a nanoplatelet morphology with the platelets (*i.e.* grains) ranging from 0.5–1 μm (BiOI) and at ∼1 μm (BiOCl) while having a thickness within the nano regime (50 and 250 nm, respectively). The BiOBr sample however consists of clusters of crystallites contributing to the particles resembling nanoflowers. The clusters were around 1 μm in diameter and appear to be composed of smaller crystallites. As mentioned before, the electronic properties of these materials are anisotropic and this faceted growth may help charge carrier mobility along certain facets. Optical spectra across the UV, visible and near IR regions were collected for the BiOX thin films and the absorbance data is plotted from 300 nm to 1100 nm in Fig. 5a. As shown, the absorption edge decreases in energy with halide anion going down group 17. Fig. 5b shows the Tauc plot of (*αhν*)^1/2^*versus* photon energy (*hν*) for BiOX (X = Cl, Br, I). Note the use of the indirect Tauc relation. It can be seen that BiOCl exhibits the largest indirect bandgap of around 3.3 eV, within the UV region. For BiOBr however, the indirect bandgap red shifts to a value of 2.7 eV (corresponding to 460 nm). Moving to BiOI, the bandgap shifts further into the visible region with a value of around 1.8 eV (690 nm light). These values are in excellent agreement with reported literature on these materials prepared by other routes.^30^,^52^,^53^

**Fig. 6:**
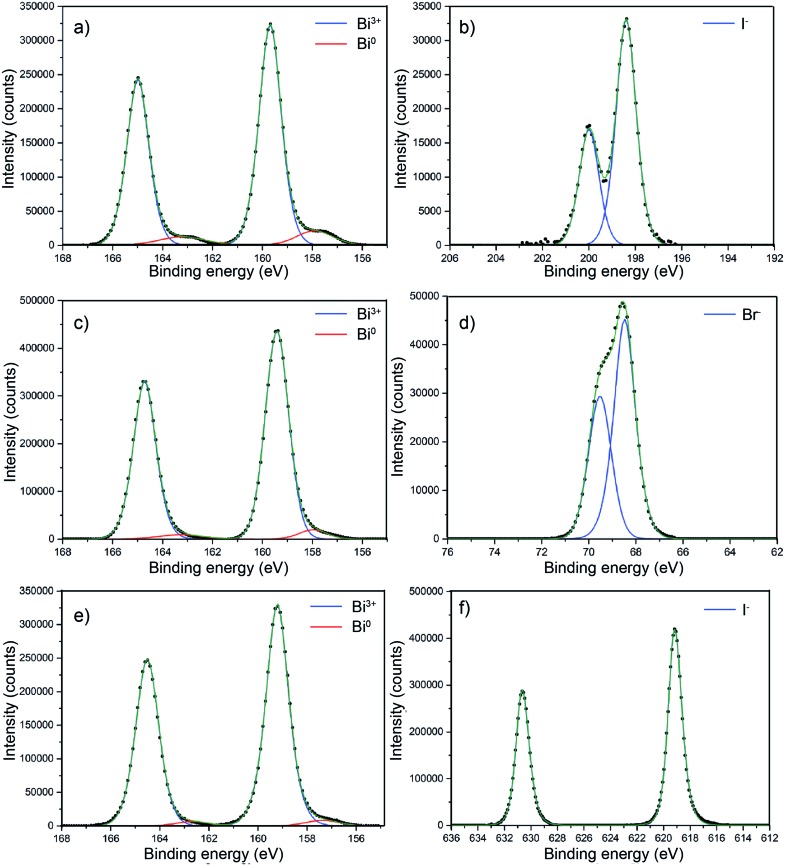
Core level X-ray photoemission spectra of (a) BiOCl on FTO glass showing the Bi^3+^ 4f_7/2_ and 4f_5/2_ transitions, with a minor Bi^(0)^ component at a lower binding energy. (b) Cl^–^ 2p_3/2_ and 2p_1/2_ transitions are also shown. (c) BiOBr on FTO glass showing the Bi^3+^ 4f_7/2_ and 4f_5/2_ transitions with a minor Bi^(0)^ component at a lower binding energy, along with the corresponding Br^–^ 3d_5/2_ and 3d_3/2_ transitions (d). (e) BiOI on FTO glass showing the Bi^3+^ 4f_7/2_ and 4f_5/2_ transitions with a minor Bi^(0)^ component at a lower binding energy, with I^–^ 3d_5/2_ and 3d_3/2_ transitions (f).

BiOX core level X-ray photoemission spectroscopy (XPS) was performed on all of the samples as shown in Fig. 6 to probe the surface composition. Fig. 6a shows the core level XPS for BiOCl. For clarity, only the metal and halide transitions are shown. The binding energy for the Bi 4f_7/2_ transition at 159.7 eV clearly corresponds to Bi^3+^.^54^,^55^ There is a minor metallic bismuth (Bi^0^) component corresponding to a Bi 4f_7/2_ transition at a binding energy of 157.9 eV. The Cl^–^ 2p_3/2_ and 2p_1/2_ transitions were observed at binding energies of 198.4 eV and 200 eV, respectively. This metallic bismuth component has been observed in many reports but is often not discussed. The nature of metallic bismuth could well be due to instability of the material under incident X-rays resulting in photoreduction to metallic bismuth. This has been observed before in bismuth based materials and also in similar compounds such as CH_3_NH_3_PbI_3_ where a metallic lead signal is found.^56^,^57^ Fig. 6c shows the core level XPS for BiOBr. The binding energy for the Bi 4f_7/2_ transition at 159.4 eV clearly corresponds to Bi^3+^.^58^ There also appears to be a minor metallic bismuth component in this sample but it is significantly reduced in comparison to the BiOCl film. Again, the least-squares fit is markedly improved with metallic bismuth components included in the model. The bromide peaks correspond to binding energies of Br^–^ and agree well with literature values.^58^Fig. 6e shows the core level XPS for BiOI. The binding energy for the Bi 4f_7/2_ transition at 159.2 eV matchs with Bi in the 3+ oxidation state.^59^ Similar to BiOBr, the metallic bismuth component is less than in the case of BiOCl. The binding energy of the iodide transitions are consistent with the literature.^59^

### Electronic structure

**Fig. 7:**
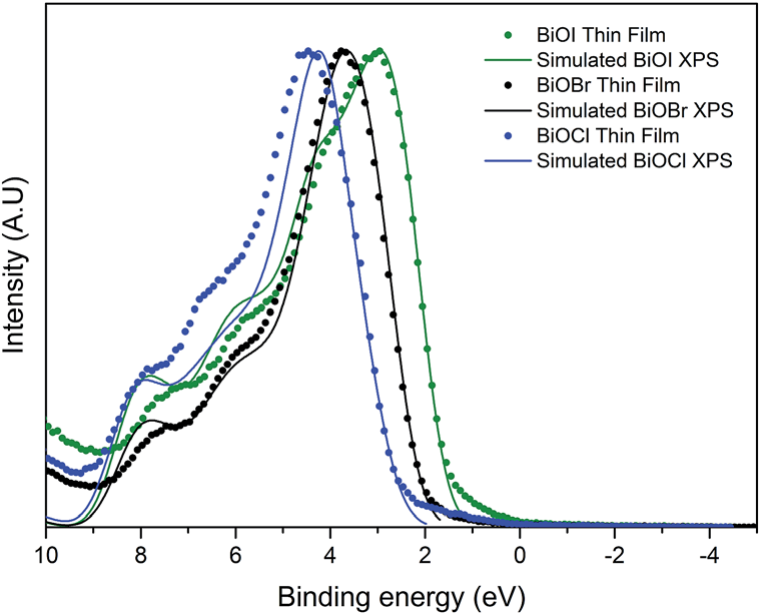
Normalized valence-band photoemission spectra of the series of BiOX films prepared by AACVD, compared with HSE06 + D3 + SOC simulated valence band XPS. Note the relative shift to a lower binding energy from BiOCl to BiOI showing band gap narrowing as you move down group 17 from Cl^–^ to I^–^.

The valence band maximum for BiOX crystals is known to be comprised of O 2p orbitals and X *n*p states (*n* = 3, 4, 5 and X = Cl, Br and I, respectively). The conduction band minimum is mainly comprised of Bi 6p states. As X gets larger, the contribution of X *n*s states increases. This results in a narrowing of the bandgap as seen experimentally through valence band XPS and optical data as well as theoretically through DFT calculations. The predicted values of the bandgaps of BiOCl, BiOBr and BiOI are *ca.* 3.4, 2.8 and 2.0 eV, although it should be noted that these do not include thermal effects. Valence band X-ray photoemission spectra are shown in Fig. 7. To understand the orbital make-up of the valence band and further confirm that the correct material had been deposited, we have compared the XPS spectra to simulated XPS VB spectra calculated using the HSE06 functional with relativistic corrections (spin–orbit coupling). The simulated XPS VB spectra were obtained by scaling the calculated partial density of states using atomic orbital photoionisation cross-sections^60^ and broadened using a 0.47 eV Gaussian function to simulate experimental broadening. The spectra are dominated by a broad valence band of O 2p states extending from the valence band onset to about 6–8 eV binding energy, in excellent agreement with the calculated electronic structure. By simple linear extrapolation, the valence band edge for BiOI occurs at around 1.6 eV; for BiOBr occurs at around 2.2 eV and for BiOCl occurs at around 2.9 eV. However, the absolute valence band edge positions cannot simply be derived by linear extrapolation of the rising edge of the valence band to the baseline. According to the DFT calculations it has been shown that there is a rapid onset of the valence band density of states leading to an almost flat band. However, due to broadening of lifetime and instrument effects of the spectrometer, a significant slope to the measured onset of the valence band was seen. Therefore, extrapolating to the baseline results in a large underestimation in the VBM to surface Fermi level separation. The relative change in positions is in agreement with optical absorbance data.

### Photoelectrochemical testing

**Fig. 8:**
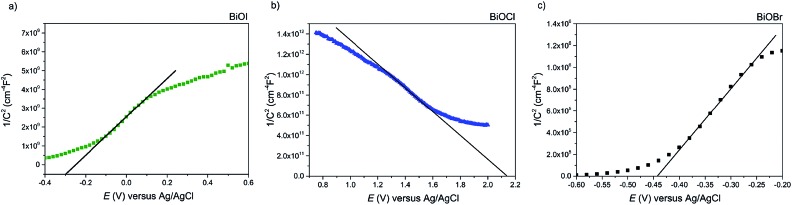
The Mott–Schottky plot for the (a) BiOI, (b) BiOCl and (c) BiOBr films grown *via* AACVD. The BiOI and BiOBr films' n-type behavior is apparent from the negative slope of the curve. The BiOCl film showed p-type properties.

Firstly, Mott–Schottky plots (impedance spectra) were recorded to investigate whether the as-synthesised materials exhibited n- or p-type behaviour and to determine the flat-band potentials. From Fig. 8a, the M–S plot for BiOI displays n-type behaviour, due to the negative slope which, when extrapolated to the *x*-axis, yields a flat-band value of –0.3 V *vs.* Ag/AgCl (+0.58 V *vs.* RHE). Taking the typical difference between the conduction and flat-band potentials to be *ca.* 0.3 V, the conduction band edge may be approximated to lie at +0.3 V *vs.* RHE.^61^ This is similar to the value reported by Mullins for spray-deposited BiOI films that also displayed n-type behaviour.^51^ Given that the measured indirect bandgaps of our BiOI films is 1.7 eV, the location of the valence band edge would correspond to 2 eV *vs.* RHE, indicating that the material could be utilised for photo-assisted water oxidation but not reduction. For AACVD-grown BiOCl films, the M–S plot revealed p-type behaviour due to the negative direction of the slope as shown in Fig. 8b.

The flat-band position was calculated to be 2.15 V *vs.* Ag/AgCl (2.73 V *vs.* RHE), therefore the valence band position equates to roughly 2.43 V *vs.* RHE, and given that the bandgap of our BiOCl is 3.2 eV, the CBE position is –0.77 V *vs.* RHE. This suggests that BiOCl could be utilised as a potential photocathode for water splitting. The M–S plot for the BiOBr film reveals n-type behaviour (Fig. 8c), with a flat-band potential of –0.44 V *vs.* Ag/AgCl (0.14 V *vs.* RHE), therefore a conduction band position of –0.16 V *vs.* RHE, in good agreement with previous reports.^62^ A valence band position of 2.44 V was calculated based on the bandgap value of 2.6 eV.

**Fig. 9:**
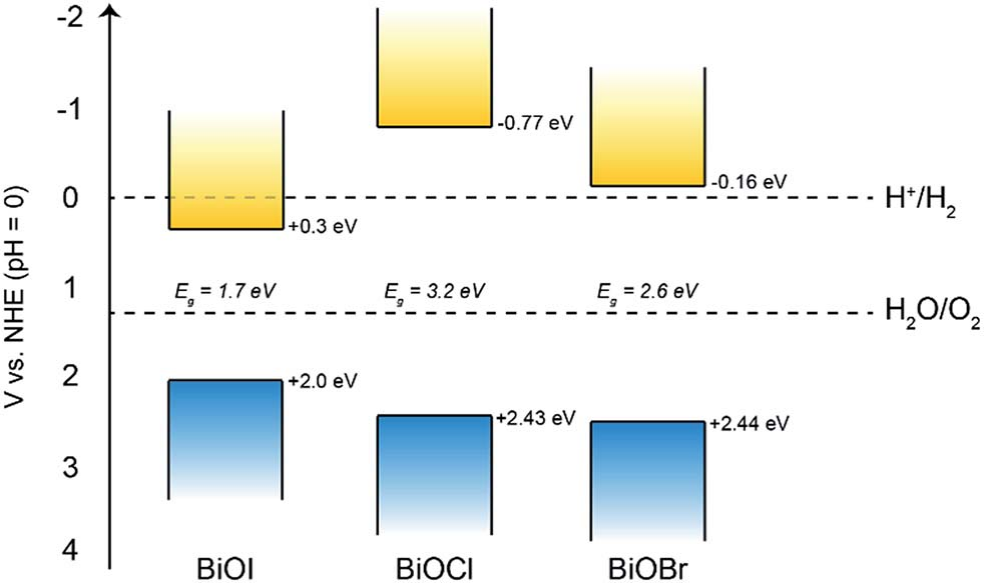
The band positions of the bismuth oxyhalide films compared to the potentials of water splitting at pH 0. Bandgap values of 1.7, 3.2 and 2.6 eV were determined for the BiOI, BiOCl and BiOBr films, respectively.

Therefore, we can construct a band diagram depicting the valence and conduction band positions of several bismuth oxyhalides with respect to the potentials of water splitting (at pH 0), see Fig. 9. Note the calculated CB minima for our AACVD grown BiOCl and BiOI film is more negative than the H_2_ evolution potential, this has been previously reported in the literature.^62^–^64^

A recent review paper highlighted that bismuth oxyhalides exhibit appreciable activity for organic decomposition, however it was also mentioned that they have been rarely used for solar fuel processes, such as water splitting and CO_2_ photoreduction.^65^ To address this and to gain a fuller understanding of their photophysical properties, we tested the BiOX films synthesised *via* AACVD for photoelectrochemical properties under 100 mW cm^–2^ illumination in 0.5 M Na_2_SO_4_ electrolyte. The current–voltage plot for the BiOCl film shows some photocathodic activity compared to the dark current, exhibiting a current of *ca.* –1 mA cm^–2^ at –0.4 V (*vs.* Ag/AgCl), however a very small anodic current was observed, approximately 0.1 mA cm^–2^ at 1.2 V (*vs.* Ag/AgCl) (see ESI†). This is somewhat in agreement with the measured flat-band potential of the material, which suggested that it could show photocathodic activity. Furthermore, the applied bias-photon-to-current conversion efficiency (ABPE) was calculated to be 0.04%. The stability test of the same film recorded at an applied voltage of –0.4 V *vs.* Ag/AgCl is shown in the ESI.†

Altogether the stability is relatively poor; the current actually increases then decreases, most probably due to photocorrosion. The cathodic photocurrent decreases by 25% after just 10 minutes of illumination. This clearly shows that BiOCl may be unsuitable for photocatalytic applications, particularly water splitting, in the absence of a protective coating. This issue is common for other photoactive materials such as Cu_2_O, which requires typically ALD grown protective layers of ZnO and Al_2_O_3_ to help slow down corrosion in water during illumination.^66^

**Fig. 10:**
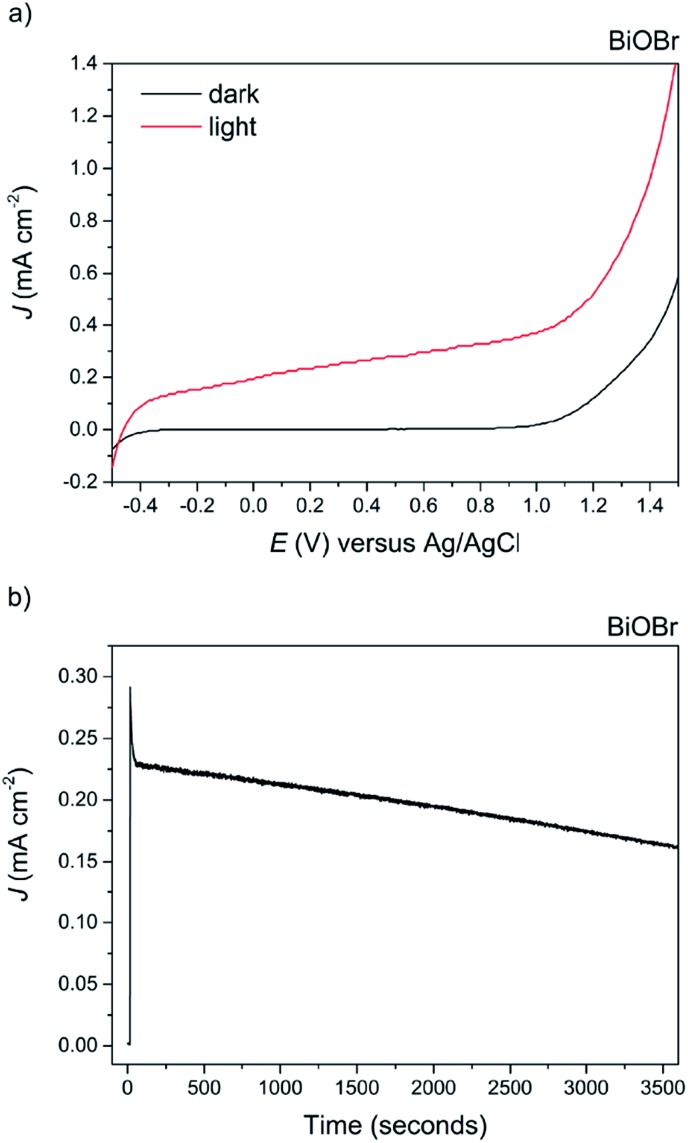
(a) The current–voltage behavior of the AACVD grown BiOBr film under 100 mW cm^–2^ illumination in 0.5 M Na_2_SO_4_ electrolyte. A photocurrent of *ca.* 0.38 mA cm^–2^ at 1 V *vs.* Ag/AgCl was observed. (b) The stability of the BiOBr film at an applied voltage of 1.0 V *vs.* Ag/AgCl. There was a 23% decrease after 1 hour.

The appearance of the BiOCl film after testing changed to black in colour, and photocorrosion to Bi metal species was confirmed *via* XRD analysis (not shown herein). Recently, the coupling of BiOCl with other metal oxides into a junction structure has resulted in improved performance for PEC water splitting; however in all such cases the performance of the bare BiOCl was either not reported or not addressed.^67^,^68^ On the other hand, photocatalytic testing of BiOBr revealed good photoanodic activity, exhibiting a photocurrent of *ca.* 0.38 mA cm^–2^ at 1 V *vs.* Ag/AgCl, see Fig. 10a. The ABPE of the BiOBr film was 0.25%.

To the best of our knowledge, this is the highest photocurrent recorded for BiOBr electrodes without the use of a sacrificial electron donor. It is also over three times higher than that recorded for BiOBr-reduced graphene composite electrodes tested under similar conditions (*ca.* 75 μA cm^–2^ at 0.45 *vs.* Ag/AgCl).^62^ Improved photocurrent and photocatalytic activity has recently been reported for (001) oriented BiOBr samples for both organic contaminant decomposition and nitrogen fixation.^63^,^69^ In fact, we find that both the (001) and (101) facets are dominant in our samples as evidenced from XRD analysis, suggesting that at least a proportion of the improved photocatalytic activity is from charge transfer and separation along the (001) facet. The stability of the material was also tested at an applied potential of 1.0 V *vs.* Ag/AgCl for 1 hour duration, revealing a *ca.* 23% decrease by the end of the experiment and no obvious change in appearance of the film (Fig. 10b). This test was for considerably longer than that reported previously in the literature. The slow photocorrosion of BiOBr is due to self-reduction to Bi metal caused by photogenerated CB electrons; the application of an external bias does alleviate this by electron transfer to the counter electrode, although not 100% efficient. The complex mechanism of photocorrosion and electrode regeneration in these BiOX materials has already been discussed,^70^ but can be summarised as follows:

3 BiOX + 2H^+^ + 3e^–^ → Bi + X^–^ + H_2_O

As our electrolyte was de-aerated, dissolved oxygen cannot act as an electron acceptor and therefore cannot protect BiOX as suggested in the above reference. Thus, we suggest a further option would be to add a passivation layer or to add a surface oxidation catalyst, which will separate surface electrons and holes and permit more efficient electron transfer to the counter electrode.^13^,^71^

**Fig. 11:**
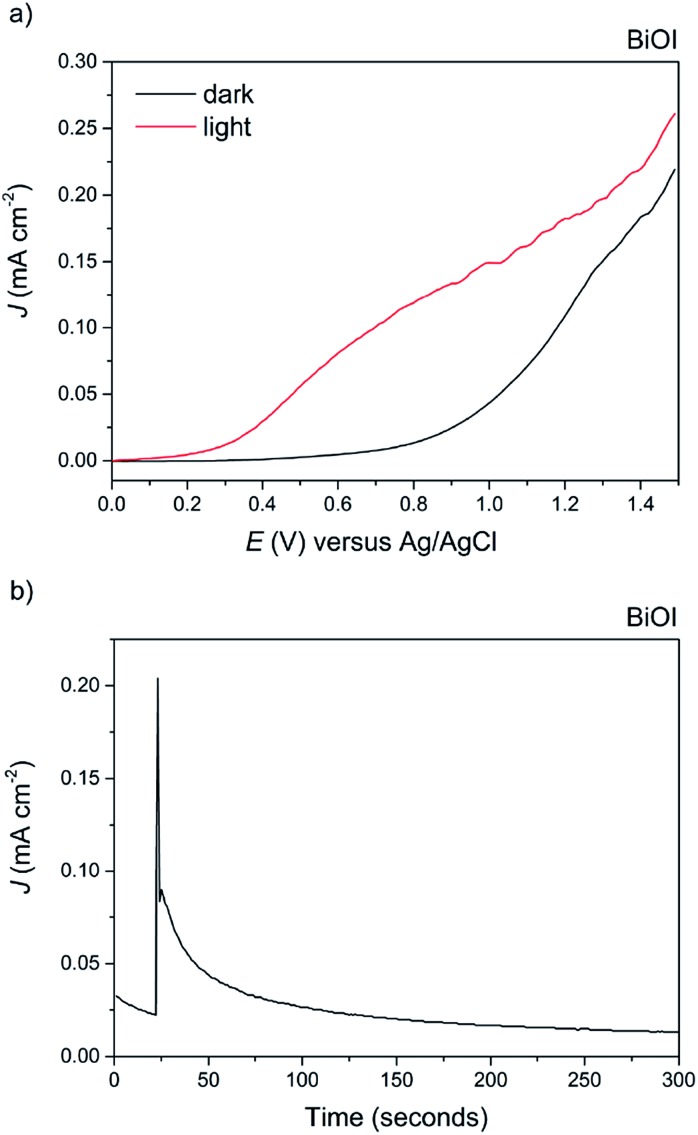
(a) The current–voltage behavior of the AACVD grown BiOI film under 100 mW cm^–2^ illumination in 0.5 M Na_2_SO_4_ electrolyte. A photocurrent of *ca.* 0.15 mA cm^–2^ at 1 V *vs.* Ag/AgCl was observed. (b) The stability of the BiOBr film at an applied voltage of 0.5 V *vs.* Ag/AgCl. There was rapid decay after just 100 seconds.

Finally, BiOI synthesized *via* AACVD was tested for its PEC performance (Fig. 11a). To the best of our knowledge, this is the first report of the photoelectrochemical activity of BiOI. Anodic behaviour was observed, in agreement with the shape of the Mott–Schottky plot, with a maximum photocurrent of 0.15 mA cm^–2^ at 1 V *vs.* Ag/AgCl and an ABPE of 0.07%. The dark current at this voltage was fairly high at 0.025 mA cm^–2^, indicative of the material's limited stability, particularly at higher applied potentials. To highlight this, a stability test was performed at an applied voltage of 0.5 V *vs.* Ag/AgCl (Fig. 11b). To our surprise, BiOI exhibited an extremely rapid decay in photocurrent, and no photocurrent was observed after a period of just 100 seconds.

Overall, it was found that the stability of BiOBr is much superior to that of both BiOI and BiOCl under the present experimental conditions, which could have important implications for their future use in photo-assisted water splitting reactions. We suggest that the enhanced water oxidation property of BiOBr is mainly due to the very positive VB position, which possesses a higher overpotential for water oxidation compared to the other BiOX materials (Fig. 9). This is in addition to the dominant (001) facet in our AACVD grown BiOBr films which allows for improved charge separation. The poor stability of BiOI is of particular concern, whilst there are several reports of this material being used for degradation of organic dyes and pollutants, it cannot be used for water splitting reactions due to its facile photo-degradation.

These results should serve as a caution for those intending to utilise untreated bismuth oxyhalides for applications in photocatalytic systems. It may prove possible to enhance stability through morphological control. Otherwise, the use of co-catalysts or protection layers should be explored in order to extend the stability of these materials towards practical applications.

## Conclusion

In conclusion, AACVD was used to grow a series of phase pure tetragonal matlockite structured BiOX (X = Cl, Br, I) films at 300 °C. SEM micrographs showed nanoplatelet morphology for BiOCl and BiOI whereas nanoflower like particles were observed for the BiOBr film. The optical bandgap calculations of the films agreed well with first-principles calculations that explained that the narrowing of the bandgap is due to the variation in the energy of the halide p orbitals that form the upper valence band. Functional testing showed that untreated BiOCl (p-type) and BiOI (n-type) were both unsuitable for photoelectrochemical applications as they showed poor stability. BiOBr revealed good photoanodic activity with a photocurrent of *ca.* 0.38 mA cm^–2^ at 1 V *vs.* Ag/AgCl – the highest photocurrent recorded for BiOBr electrodes without the use of a sacrificial electron donor. BiOBr also showed good stability, far better than BiOCl and BiOI under the testing conditions.

## Author contributions

D. S. B. designed/carried out the experiments, performed the sample characterization measurements and analyzed the results. S. J. A. M. carried out the PEC measurements and analyzed the results. D. S. B., S. J. A. M., S. S., D. O. S. and A. W. wrote the manuscript. D. S. B., S. J. A. M., S. S. and A. W. made the figures. D. O. S. and A. W. performed and analyzed the computational measurements. C. J. C. and I. P. P. supervised D. S. B. and S. S. J. T. supervised S. J. A. M. D. O. S., A. W., I. P. P., J. T. and C. J. C. were involved in the design of experiments, editing of the manuscript and discussions throughout the work. S. M. B. was involved in a number of scientific discussions and analysis of results. A. Y. O. generated ideas and spearheaded the initial collaboration between the institutions. M. M. was involved with a number of scientific discussions and analysis of results and has provided valuable insights, which have in turn, directed the research. All authors contributed to the scientific discussion.

## Supplementary Material

Supplementary information
