# Supplementary material for: Bismuth oxyhalides: synthesis, structure and photoelectrochemical activity
Source: Chem Sci. 2016 Mar 9;7(8):4832–41. doi: 10.1039/c6sc00389c (PMC6016733; doi:10.1039/c6sc00389c)
Supplement: Supplementary file 1 [file SC-007-C6SC00389C-s001.pdf]

Electronic Supplementary Material (ESI) for Chemical Science.
This journal is © The Royal Society of Chemistry 2016

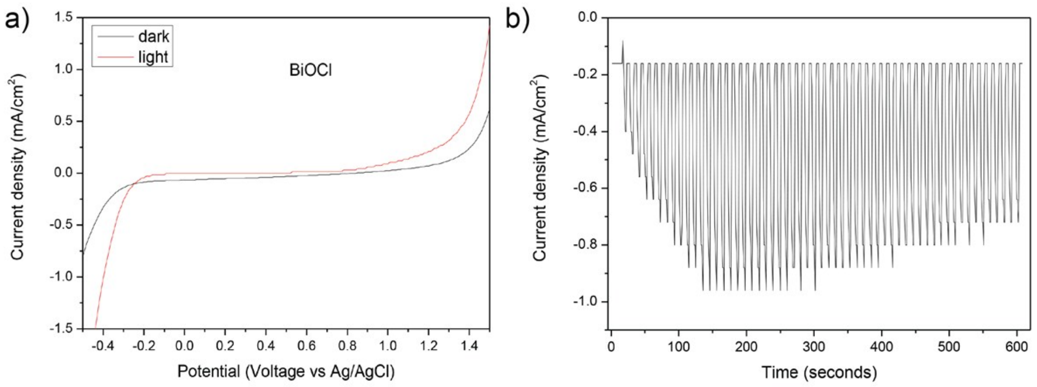

Figure 1: a) The current-voltage behavior of the AACVD grown BiOCl film under 100 mW/cm^2 illumination in 0.5M Na_2SO_4 electrolyte. b) The stability of the BiOCl film at an applied voltage of -0.4 V vs Ag/AgCl.

$$\eta/\% = \left[\frac{|J_{ph}|(mAcm^{-2}) \times (1.23 - |V_b|)/(V)}{P_{total}\,(mAcm^{-2})}\right]_{AM1.5G} \times 100$$

Equation 1: was used to determine the applied bias-photon-to-current conversion efficiency for the three BiOX films grown via AACVD.
